# Downhill Esophageal Variceal Rupture Caused by Superior Vena Cava Syndrome in NUT Carcinoma: A Case Report

**DOI:** 10.7759/cureus.107043

**Published:** 2026-04-14

**Authors:** Keisuke Okuno, Sosuke Kakee, Daisuke Kawaba, Kenji Yoshida, Noriyuki Namba

**Affiliations:** 1 Department of Pediatrics, Tottori University Hospital, Yonago, JPN; 2 Department of Radiation Oncology, Tottori University Hospital, Yonago, JPN

**Keywords:** adolescent, downhill esophageal variceal bleeding, endoscopy, nut midline carcinoma (nmc), superior vena cava (svc) syndrome

## Abstract

NUT carcinoma is a rare and aggressive malignancy that frequently arises in midline structures and is often associated with superior vena cava (SVC) syndrome. However, life-threatening complications related to venous congestion are not well characterized. Herein, we report a case of a 14-year-old boy with mediastinal NUT carcinoma who developed downhill esophageal varices with massive bleeding secondary to SVC obstruction. Despite multimodal therapy including radiation and chemotherapy, the disease progressed rapidly. Three months after admission, the patient presented with hematemesis, and endoscopy revealed bleeding esophageal varices, which were successfully treated with endoscopic ligation. This case highlights a rare but potentially fatal complication of SVC syndrome in NUT carcinoma. Clinicians should be aware of the risk of esophageal variceal formation and bleeding in patients with severe SVC obstruction.

## Introduction

NUT carcinoma is a rare and highly aggressive malignancy characterized by chromosomal rearrangements involving the NUT gene [1-2]. It typically arises in midline structures such as the mediastinum, head and neck, and thorax, and is associated with a poor prognosis. Superior vena cava (SVC) syndrome is a well-recognized complication of mediastinal tumors, including lymphoma and lung cancer, due to tumor compression or invasion of the SVC [3].

In portal hypertension caused by liver cirrhosis, varices form in the lower esophagus due to ascending collateral circulation. On the other hand, when a mediastinal tumor compresses the SVC, collateral circulation flows from the cephalad to the caudal direction in the veins surrounding the esophagus, resulting in the formation of varices primarily in the upper esophagus [4]. If compression extends to the azygos venous system, varices may also develop in the lower esophagus [4]. Although SVC syndrome is relatively common in mediastinal malignancies, bleeding from downhill esophageal varices is rare. To our knowledge, esophageal variceal rupture associated with NUT carcinoma has not been previously reported.

Herein, we describe a pediatric case of mediastinal NUT carcinoma complicated by SVC syndrome leading to downhill esophageal variceal bleeding.

## Case presentation

**Figure 1:**
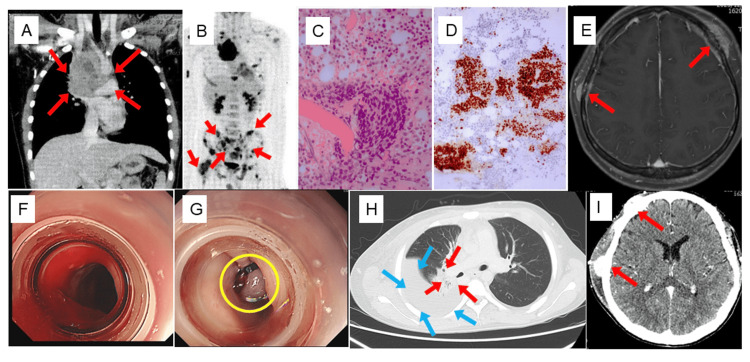
Imaging and histological findings of the case Images A through D were obtained at the previous hospital. On chest contrast-enhanced CT, a tumor was identified in the mediastinum (A), and PET-CT revealed extensive metastases throughout the skeleton, particularly the ilium and right femur (B). Biopsy of the iliac metastasis showed numerous undifferentiated tumor cells on hematoxylin-eosin stain (C: 400×). These tumor cells were positive for NUT immunohistochemistry (D: 400×). At admission to our hospital, the brain MRI suggested multiple metastases in the subcutaneous tissue of the head (E). Three months after admission, the patient experienced massive hematemesis. Emergency endoscopy revealed a variceal lesion in the mid esophagus that was bleeding (F). Endoscopic variceal ligation was performed, and hemostasis was achieved. The ligated varix is indicated by the yellow circle (G). Contrast CT of the chest (H) and contrast CT of the head (I) are shown six months after admission. Despite intensive radiation and chemotherapy, the mediastinal mass was enlarged with pleural effusion (blue arrows), and the subcutaneous mass of the head worsened. The primary tumor and metastatic lesions are indicated by the red arrows.

A 14-year-old boy presented with chest pain, dyspnea, and insomnia. Imaging studies revealed a large mass in the superior mediastinum compressing the superior vena cava and airway, along with multiple bone metastases. A biopsy of the iliac lesion demonstrated poorly differentiated carcinoma with positive immunohistochemical staining for NUT, confirming the diagnosis of NUT carcinoma (Fig. 1A-1D).

One month after symptom onset, the patient was referred to our hospital. On admission, he exhibited decreased breath sounds on the right side and palpable subcutaneous masses on the scalp. Blood tests revealed abnormal coagulation, elevated lactate dehydrogenase (1,279 IU/L), and C-reactive protein (13.4 mg/dL) levels. Imaging confirmed extensive metastatic disease, including lesions in the subcutaneous tissues of the head (Fig. 1E).

**Figure 2:**
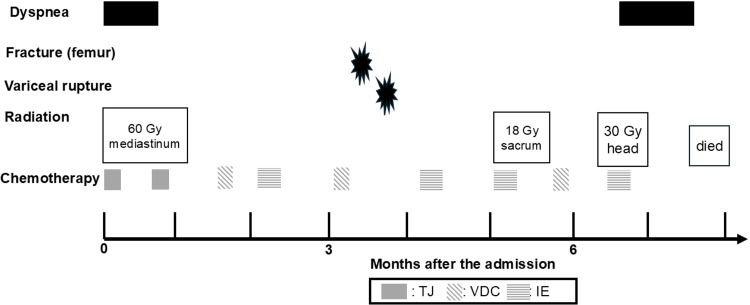
Clinical course TC, paclitaxel 200 mg/m^2^ and carboplatin; target area under the curve, 5 mg/mL/min on day 1; VDC, vincristine 1.5 mg/m^2^, doxorubicin (DOX) 37.5 mg/m^2^, and cyclophosphamide 1.2 g/m^2^ on day 1 (DOX is also administered on day 2); IE, ifosfamide 1.8 g/m^2^, and  etoposide 100 mg/m^2^ on days 1-5.

Initial treatment consisted of radiotherapy (60 Gy) to the mediastinal tumor combined with paclitaxel and carboplatin chemotherapy (TJ). Although the mediastinal mass decreased in size and respiratory symptoms improved, bone metastases progressed. Subsequent treatment with vincristine, doxorubicin, cyclophosphamide, ifosfamide, and etoposide (VDC-IE) was ineffective (Fig. 2).

Although zoledronic acid and denosumab were administered for bone metastasis on admission, there was no improvement in the pain. Therefore, oxycodone, nonsteroidal anti-inflammatory drugs, and acetaminophen were required for pain control throughout the course of treatment.

We performed cancer genomic profiling by liquid biopsy with FoundationOne® Liquid CDx (Foundation Medicine, Inc., Cambridge, MA, USA; distributed by Chugai Pharmaceutical Co., Ltd., Tokyo, Japan). Although genetic rearrangements of the oncogene BRD4 (MSML3::BRD4 and BRD4::NOP10) were observed, no actionable findings for companion diagnostics were identified. The representative genetic rearrangement of NUT carcinoma is BRD4::NUT, but it was not detected in the current case [5]. Due to insufficient tumor tissue, genomic profiling of the tumor tissue could not be performed.

Three months after admission, the patient developed a pathological fracture of the right femur, followed by sudden hematemesis. Emergency upper gastrointestinal endoscopy revealed actively bleeding esophageal varices in the mid-esophagus (Fig. 1F). Endoscopic variceal ligation was successfully performed, achieving hemostasis (Fig. 1G). The obstruction of the SVC likely caused increased venous pressure in collateral pathways, including the azygos and esophageal venous systems.

Despite continued chemotherapy and palliative radiotherapy for metastatic lesions, the disease progressed. The tumor in the right mediastinum had regrown, and there was again pleural effusion on the same side as well as worsening of the subcutaneous mass on the head; furthermore, there was no response to chemotherapy (Fig. 1H-1I). The patient died eight months after diagnosis.

## Discussion

This case illustrates a rare but clinically significant complication of SVC syndrome in NUT carcinoma: downhill esophageal variceal bleeding.

**Figure 3:**
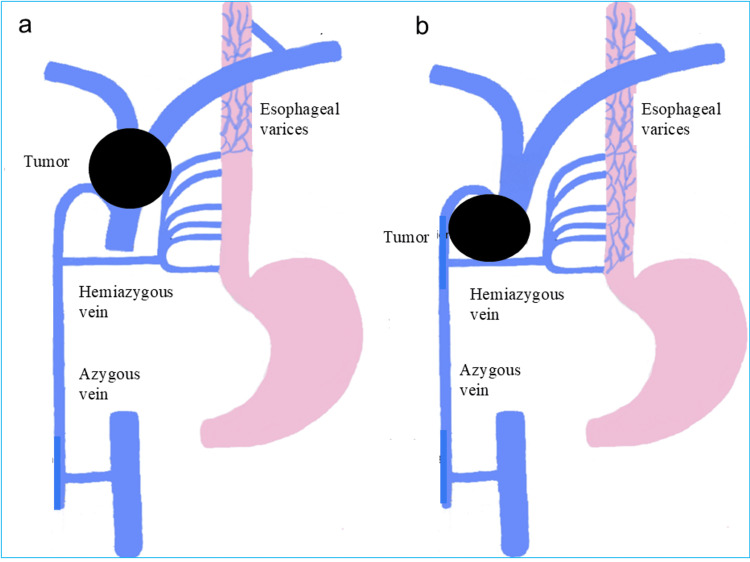
Mechanism of tumor-induced descending esophageal varices (a) Obstruction at the level of the superior vena cava (SVC). (b) Obstruction extending to the SVC and the azygos vein system. This figure was created by modifying the original figure from [4]. This figure was not generated by AI, but was created by the authors editing text in PowerPoint and then converting it to TIFF. The TIFF image was further formatted using Microsoft Photo (Microsoft Corp., USA).

As shown in Fig. 3, downhill esophageal varices develop due to obstruction of venous return in the SVC system, leading to increased pressure in collateral pathways such as the azygos vein and esophageal venous plexus [4]. Unlike portal hypertension-related varices, which occur in the lower esophagus, downhill varices typically form in the upper or mid-esophagus [4]. Although SVC syndrome is frequently observed in mediastinal tumors, only a small proportion of patients with SVC syndrome develop clinically significant variceal bleeding [3,6]. Only 14% of cases of SVC syndrome caused by tumors result in esophageal bleeding [7].

The most common cause of descending esophageal varices resulting from SVC obstruction is iatrogenic factors such as dialysis catheters; malignant tumors are a rare cause [8]. Reports of tumor-associated downhill variceal bleeding are extremely limited, particularly in pediatric patients. To the best of our knowledge, there have been no reports of descending esophageal varices or bleeding caused by NUT carcinoma. The mediastinal mass in this case was large and may have compressed not only the superior vena cava but also the azygos vein system. As shown in Figure 3b, we believe that the varices formed in the mid-esophagus, rather than in the upper esophagus, via collateral circulation from the hemiazygos vein.

In the present case, a contrast-enhanced CT scan obtained after bleeding from an esophageal variceal rupture failed to identify any shunt vessels. Importantly, the bleeding occurred despite initial tumor shrinkage following radiotherapy, suggesting that partial relief of SVC obstruction may not be sufficient to prevent complications. Upper gastrointestinal endoscopy is currently the only effective method available for diagnosing and treating downhill esophageal varices. For example, monthly or bimonthly endoscopies may enable the early detection and monitoring of varices, as well as the prevention of bleeding.

NUT carcinoma remains a highly aggressive disease with no established standard treatment, particularly in unresectable cases. Although multimodal therapy may provide temporary symptom relief, the prognosis remains poor [9-12]. Therefore, early recognition and management of complications are essential to improve patient outcomes and quality of life. Molecularly targeted drugs such as the Bromodomain and Extra-Terminal domain (BET) inhibitors [13], histone deacetylase inhibitors (HDAC) [14], and immune checkpoint inhibitors [15] have been used only in a small number of cases, and their effectiveness was limited.

This case provides several important clinical implications. First, clinicians should consider the possibility of esophageal varices in patients with SVC syndrome, especially when symptoms such as dysphagia or hematemesis occur. Second, early endoscopic evaluation may be warranted in selected cases with severe SVC obstruction. Third, standard oncologic treatments may not adequately prevent complications related to venous congestion, highlighting the need for careful monitoring.

## Conclusions

We report a rare case of downhill esophageal variceal rupture caused by SVC syndrome in a patient with mediastinal NUT carcinoma. In addition to the primary tumor in the mediastinum, numerous metastases were observed from the outset. The tumor was resistant to both radiation therapy and chemotherapy, and the patient suddenly developed a descending esophageal variceal rupture. This case highlights the importance of recognizing potentially fatal complications associated with SVC obstruction. Awareness of this condition may facilitate early diagnosis and appropriate management through early endoscopic intervention.
